# Evaluation of the combination of the dual m-TORC1/2 inhibitor vistusertib (AZD2014) and paclitaxel in ovarian cancer models

**DOI:** 10.18632/oncotarget.23022

**Published:** 2017-12-06

**Authors:** Anne-Christine Wong Te Fong, Parames Thavasu, Sladjana Gagrica, Karen E. Swales, Martin O. Leach, Sabina C. Cosulich, Yuen-Li Chung, Udai Banerji

**Affiliations:** ^1^ Cancer Research UK Cancer Imaging Centre, Division of Radiotherapy and Imaging, The Institute of Cancer Research and The Royal Marsden, London, UK; ^2^ Division of Cancer Therapeutics, The Institute of Cancer Research, London, UK; ^3^ IMED Oncology, AstraZeneca, Cancer Research UK Cambridge Institute, Cambridge, UK; ^4^ Division of Cancer Therapeutics and Division of Clinical Studies, The Institute of Cancer Research and The Royal Marsden, London, UK

**Keywords:** mTORC1/2, vistusertib, AZD2014, chemo-resistance, ovarian cancer

## Abstract

Activation of the PI3K/mTOR pathway has been shown to be correlated with resistance to chemotherapy in ovarian cancer. We aimed to investigate the effects of combining inhibition of mTORC1 and 2 using the mTOR kinase inhibitor vistusertib (AZD2014) with paclitaxel in *in vitro* and *in vivo* ovarian cancer models. The combination of vistusertib and paclitaxel on cell growth was additive in a majority of cell lines in the panel (*n =* 12) studied. A cisplatin- resistant model (A2780Cis) was studied *in vitro* and *in vivo*. We demonstrated inhibition of mTORC1 and mTORC2 by vistusertib and the combination by showing reduction in p-S6 and p-AKT levels, respectively. In the A2780CisR xenograft model compared to control, there was a significant reduction in tumor volumes (*p* = 0.03) caused by the combination and not paclitaxel or vistusertib alone. *In vivo*, we observed a significant increase in apoptosis (cleaved PARP measured by immunohistochemistry; *p* = 0.0003). Decreases in phospholipid and bioenergetic metabolites were studied using magnetic resonance spectroscopy and significant changes in phosphocholine (*p* = 0.01), and ATP (*p* = 0.04) were seen in tumors treated with the combination when compared to vehicle-control. Based on this data, a clinical trial evaluating the combination of paclitaxel and vistusertib has been initiated (NCT02193633). Interestingly, treatment of ovarian cancer patients with paclitaxel caused an increase in p-AKT levels in platelet-rich plasma and it was possible to abrogate this increase with the co-treatment with vistusertib in 4/5 patients: we believe this combination will benefit patients with ovarian cancer.

## INTRODUCTION

Over 225,000 patients are diagnosed with ovarian cancer each year, with more than 14,200 deaths reported annually worldwide [1]. The most common form of ovarian cancer is high grade serous ovarian cancer; other important subtypes include clear cell and low grade ovarian cancer.

Current treatment options include surgery followed by chemotherapy and if metastatic patient survival is approximately 3–4 years. The most frequently used chemotherapeutic agents for treatment include platinum agents (cisplatin/carboplatin) [2], paclitaxel [3] and liposomal doxorubicin [4]. Targeted agents have been introduced into the care of patients with ovarian cancer with the registration of antibodies against VEGF: bevacizumab [5] and PARP inhibitors [6].

Despite initial responses to chemotherapy in the metastatic setting, chemo-resistance is inevitable. Multiple mechanisms of resistance to chemotherapy have been explored and activation of the PI3K/mTOR pathway has been shown to be associated with resistance to chemotherapy [7, 8]. Our laboratory quantified the levels of phosphorylation of S6K, AKT and GSK3ß in ovarian cancer cells isolated from ascites. The levels of phosphorylation of S6K were found to be correlated with resistance to subsequent chemotherapy in patients who were chemo-naïve and those who had had previous chemotherapy, while the levels of phosphorylation of AKT were correlated with resistance to future chemotherapy if the patients were chemo-naïve. Other groups have also shown that activation of the PI3K/mTOR pathway is correlated with resistance to chemotherapy in ovarian cancer [9].

Building on this body of data, we hypothesized that inhibiting mTORC1 and 2 (therefore decreasing the levels of p-AKT and p-S6K) in combination with chemotherapy may improve the outcomes of patients with ovarian cancer. We planned to investigate a combination of paclitaxel and the mTORC1/2 inhibitor vistusertib in our experiments. We chose paclitaxel because it is used in the treatment of advanced ovarian cancer in weekly schedules, allowing co-administration and interaction with vistusertib on a weekly basis. Vistusertib is a well characterized mTORC1/2 inhibitor which inhibits signaling of S6K by inhibiting mTORC1, and AKT by inhibiting mTORC2 [10–12].

We aimed to: a) study the effects of growth on a panel of ovarian cancer cell lines following treatment with paclitaxel, vistusertib and the combination; b) examine the effects of signaling and apoptosis of the combination in a cisplatin-resistant cell line model; c) investigate the effects of paclitaxel, vistusertib and the combination treatment on tumors *in vivo* to gain mechanistic insights into growth, apoptosis, angiogenesis and tumor metabolism; d) to recapitulate any effects seen in human tissue in patients treated with paclitaxel and vistusertib.

## RESULTS

### Growth inhibition

**Figure 1:**
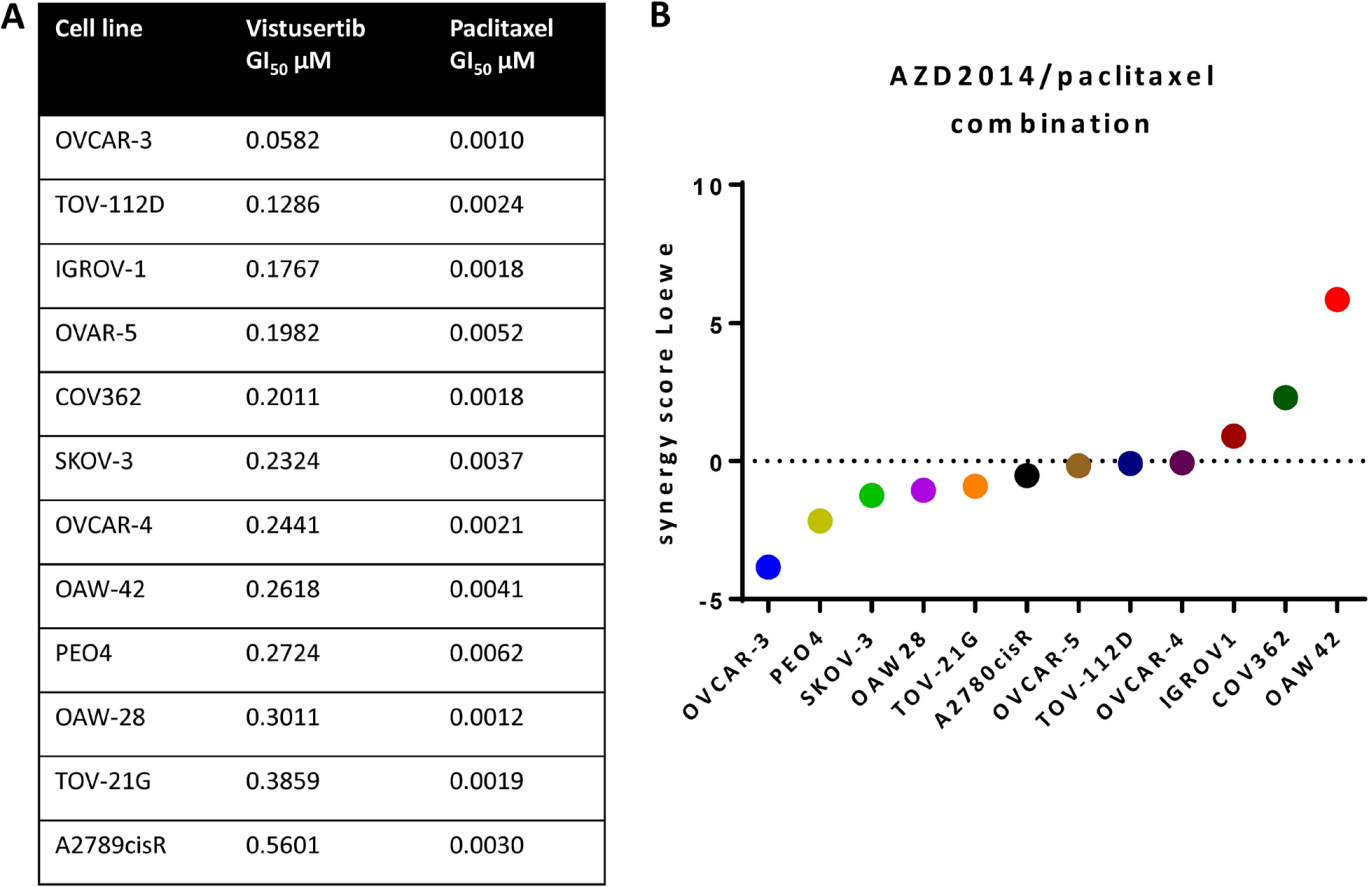
Growth inhibition by vistusertib and paclitaxel in a human ovarian cancer cell line panel (**A**) GI_50_ of vistusertib and paclitaxel in ovarian cancer cell lines. (**B**) Synergy score using Loewe model of additivity. Only one cell line showed a synergy score of > 5.

Vistusertib and paclitaxel caused growth inhibition across a panel of 12 human ovarian cancer cell lines (Figure 1A). Using a quantitative synergy score based on the Loewe model of additivity and a cut-off synergy score of > 5, one cell line (OAW42) showed synergistic growth inhibition of the concomitant combination of vistusertib and paclitaxel and the rest showed additive effects on growth (Figure 1B).

### Changes in signaling

**Figure 2:**
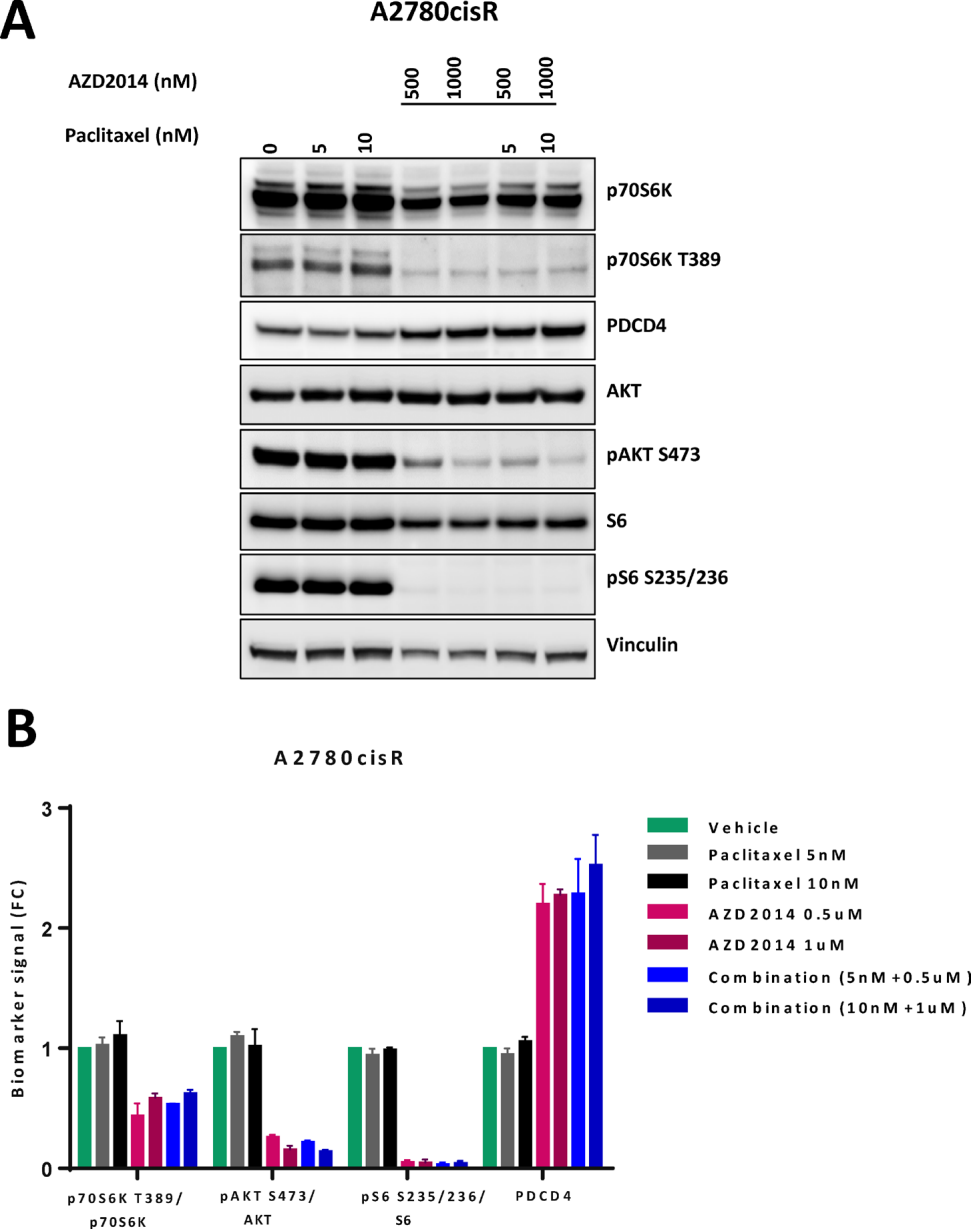
Effects of vistusertib and paclitaxel on signal transduction (**A**) Cells were exposed to paclitaxel (5 nM, 10 nM), vistusertib (500 nM, 1000 nM) or both for 24 h. Figure representative of western blot analysis was done in duplicate. Total protein was normalized to vinculin as loading control while each phosphoprotein was normalized to their total protein. There is reduction in levels of p-S6K, p-S6 and p-AKT and an induction in PCD4. (**B**) Quantification of protein expression using Syngene software showing mean of two experiments.

We chose to study A2780cis as it represented the cisplatin resistance seen in advanced ovarian cancer and was representative of the median of the synergy index in the panel of ovarian cancer cell lines studied. We quantified p-S6K (S235/236) and p-AKT (S473) levels in cells exposed to paclitaxel and vistusertib for 24 h. There was robust inhibition of signaling to targets of mTORC1 (S6K, S6) and mTORC2 (AKT) in A2780Cis treated with vistusertib or the combination of paclitaxel and vistusertib. In addition, we observed induction of the tumor suppressor protein PDCD4 (programed cell death protein 4), which is negatively regulated by S6K, confirming robust inhibition of S6K activity in this model (Figure 2).

### Evaluation of the combination *in vivo*

**Figure 3:**
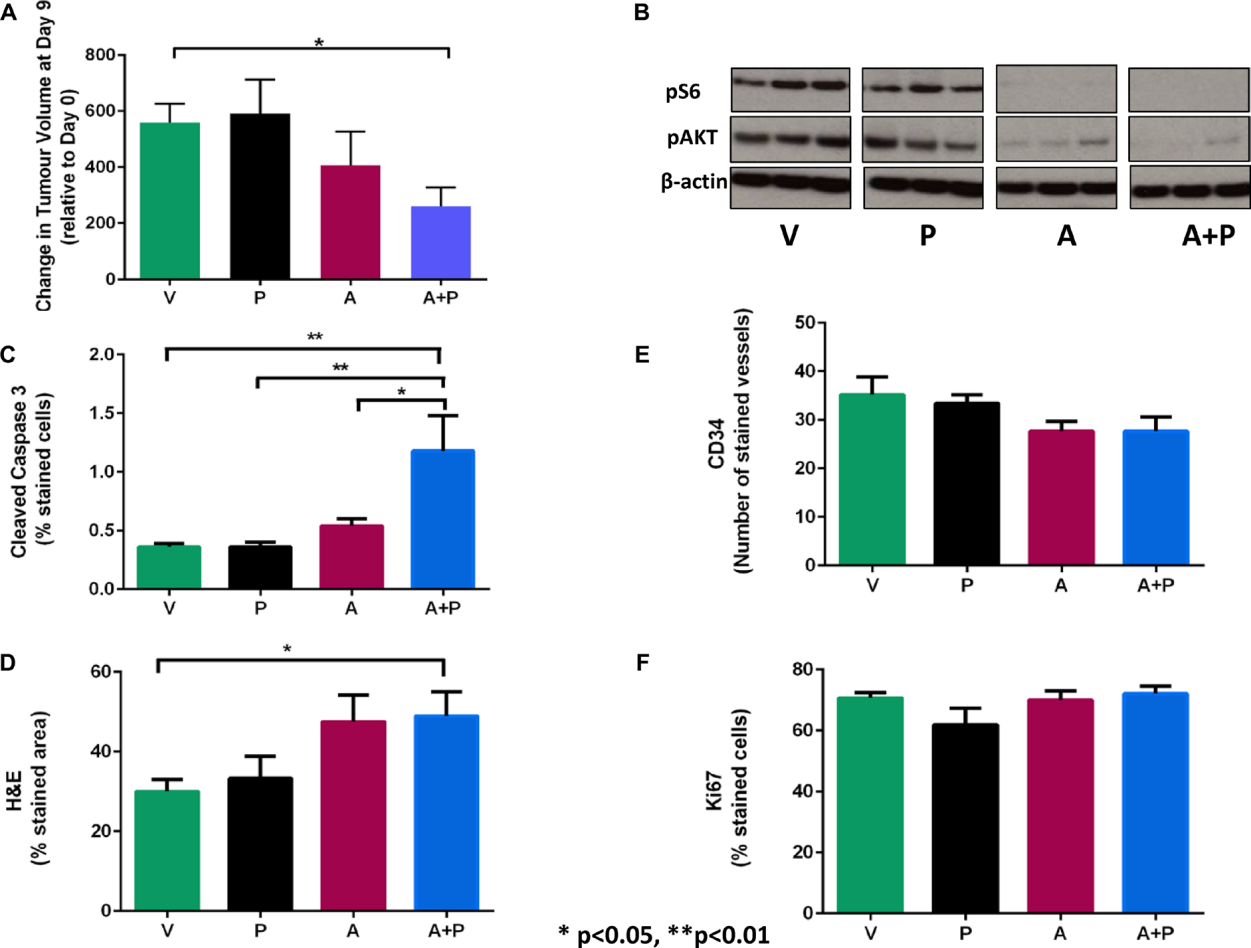
Growth and molecular response of A2780CisR xenografts following 2 weeks of treatment with vistusertib (A) alone, paclitaxel (P) alone, the combination of vistusertib and paclitaxel (A+P) and vehicle (V). (**A**) Changes in tumor volume after 2 weeks of treatment. (**B**) western blots of pSer^473^-AKT and p-Ser^240/244^-S6 protein and β-actin (loading control). (**C**)Immunohistochemistry was used to stain cleaved Caspase-3 for apoptosis. (**D**) H&E for necrosis (**E**), CD34 for microvessel density. (**F**) Ki67 for proliferation . Data are expressed as median and interquartile ranges (25% and 75%). ^*^p < 0.05 and ^***^p < 0.001; *n* = 9 in each treatment group.

We chose to evaluate the combination of vistusertib and paclitaxel *in vivo* in an A2780cis xenograft model. A2780Cis xenograft-bearing mice were treated with a vehicle control (V), vistusertib (A), paclitaxel (P) and the combination of vistusertib and paclitaxel (A + P). Following treatment for two weeks, tumors grew in all four arms: V arm with a median of 480% (25% and 75 % interquartile ranges (IQR) of 420–660), A 250% (IQR 160–640), P 490% (IQR 300–940), A + P 220% (IQR 100–370). There was a statistically significant difference between the volumes of tumor in the A + P group *vs* the V group (*p* = 0.03; Figure 3A). Reduced p-AKT (S473) and p-S6 (S240/244) levels were observed in A and A + P-treated xenografts and not in V or P arms, consistent with mTORC1/2 inhibition (Figure 3B). Importantly, there was increased apoptosis (cleaved caspase 3 positive cells) observed in the A + P arm *vs* the V arm: 0.83 *vs* 0.34; *p* = 0.0003 (Figure 3C). Furthermore, there was an increased percentage of cells showing morphological features of necrosis in the A + P arm and the V arm: 50 *vs* 30; *p* = 0.03 (Figure 3D). A study of angiogenesis in tumors quantified as the number of vessels stained by CD34 did not show any significant differences between the A + P and any other treatment arms (Figure 3E). Studies quantifying proliferation as measured by Ki67-positive nuclei within tumors did not reveal any significant differences between the A + P and other treatment arms (Figure 3F).

**Figure 4:**
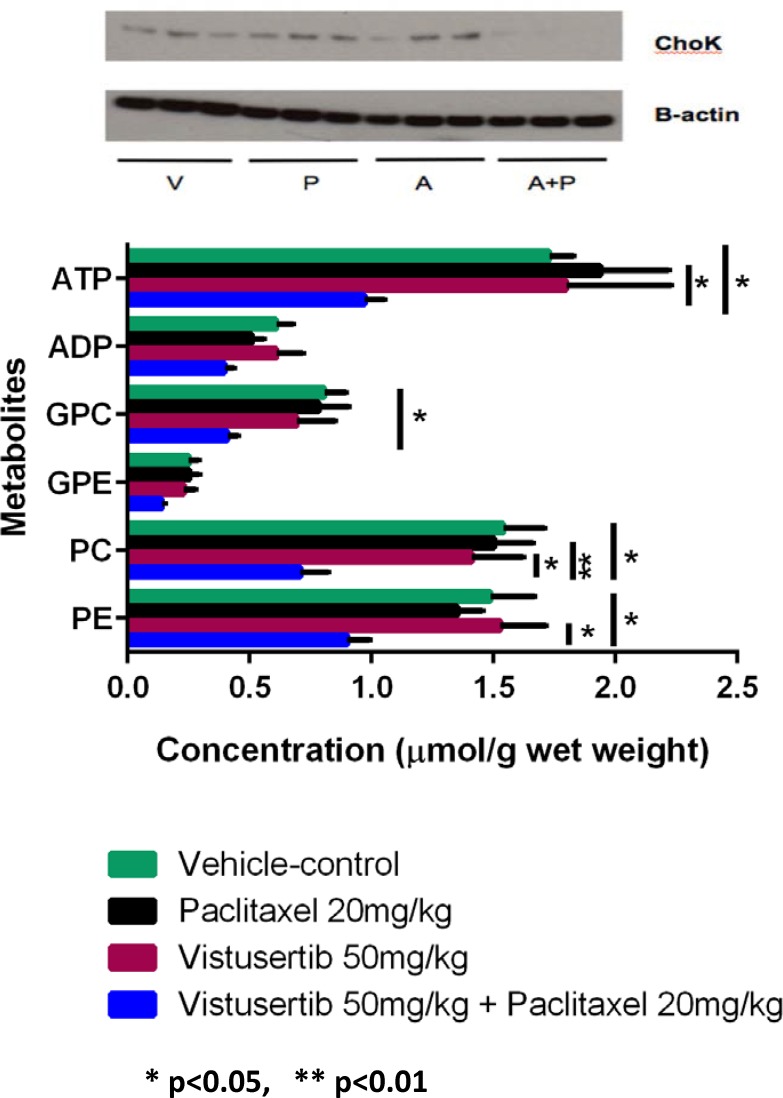
Metabolic response and choline kinase status of A2780CisR xenografts following 2 weeks of treatment with vistusertib (A) alone, paclitaxel (P) alone, the combination of vistusertib and paclitaxel (A+P) and vehicle (V). Choline kinase expressions were measured by western blotting and β-actin is used as a loading control. Tumor phospholipid and bioenergetic metabolites were measured by ^31^P-MRS of tumor extracts. Data are expressed as median and interquartile ranges (25% and 75%). ^*^ *p* < 0.05; minimum *n* = 7 in each treatment group.

To assess the effects of the different treatments on metabolism, we analyzed the A2780Cis xenograft samples using ^31^P MRS. We observed significant decreases in phosphocholine (PC; *p* = 0.01), glycerophosphocholine (GPC; *p* = 0.02), phosphoethanolamine (PE; *p* = 0.04), and adenosine triphosphate (ATP; *p* < 0.05), together with reduced expression of choline kinase in the combination-treated tumors when compared to vehicle-controls, suggesting that the combination had significant effects on phospholipid and bioenergetics metabolism (Figure 4).

### Study of changes in signaling in patients

**Figure 5:**
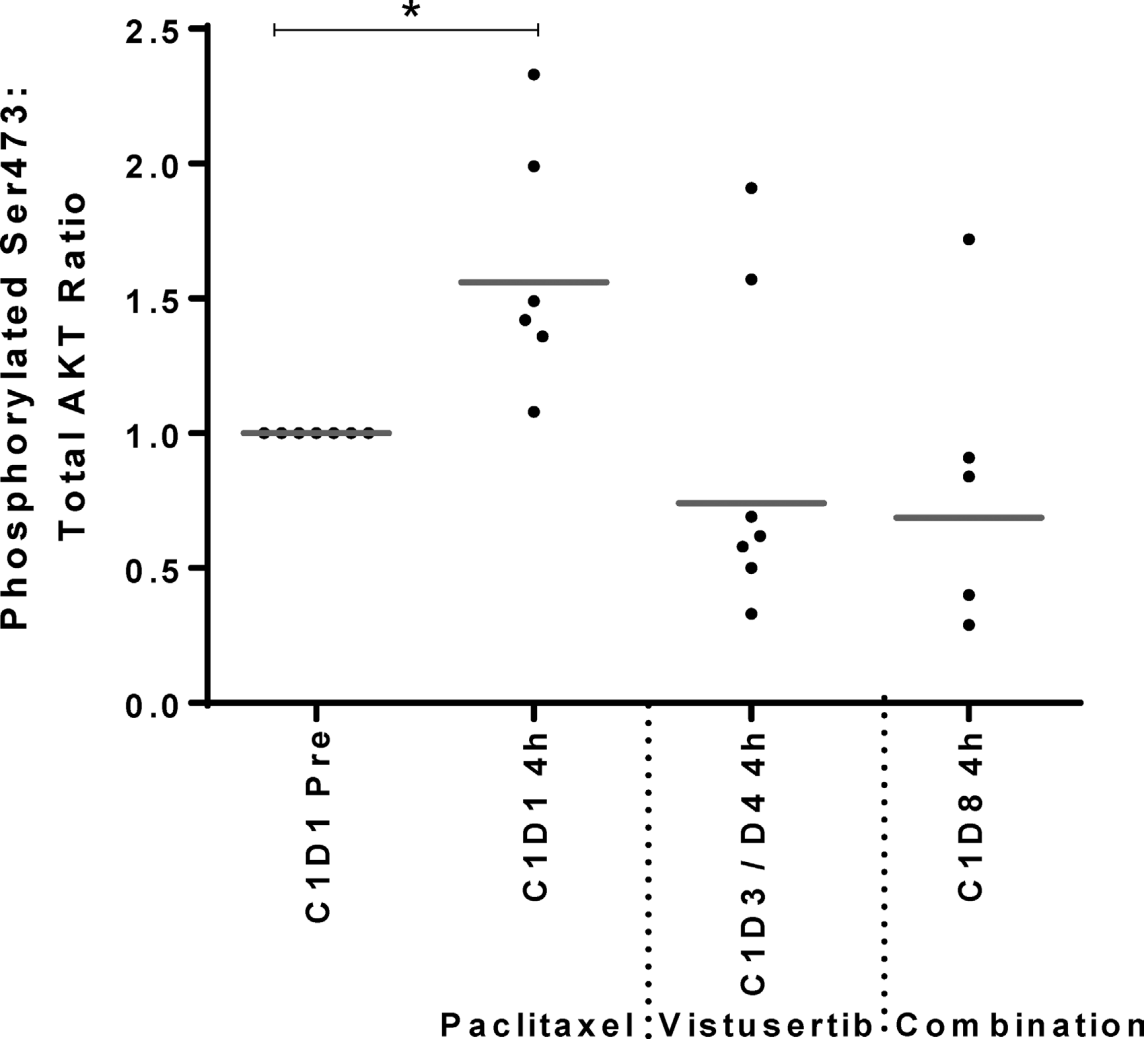
Modulation of AKT phosphorylation following 4h of treatment with paclitaxel alone on cycle 1 day 1 (C1D1 4 h), vistusertib alone on cycle 1 day 3 or 4 (C1D3/4 4 h) and the combination of vistusertib and paclitaxel on cycle 1 day 8 (C1D8 4h) compared to pre-dose (C1D1 pre) in platelet-rich plasma (PRP) of 7 ovarian cancer patients p-AKT levels in PRP were measured by MSD electrochemiluminescent immunoassay and normalized to the total AKT levels. ^*^p < 0.05 paired *t* test; minimum *n* = 5 at each time point.

Patients with ovarian cancer who received paclitaxel and vistusertib on a phase I study had platelet-rich plasma (PRP) collected at 4 h following dosing with either agent alone or in combination. Interestingly, there was a statistically significant increase in p-AKT (S473) levels following administration of paclitaxel (*p* = 0.0225) and reduction of p-AKT levels in 5 out of 7 patients following dosing with vistusertib (*p* = 0.634). The addition of vistusertib to paclitaxel reduced the p-AKT levels that were induced following paclitaxel dosing in 4 out of 5 patients (*p* = 0.0996; Figure 5).

## DISCUSSION

Paclitaxel chemotherapy has been used for the treatment of ovarian cancer for more than three decades. While the majority of its use is in combination with platinum chemotherapy in a three weekly schedule [3, 13], weekly administration of paclitaxel, often in patients who have received prior 3-weekly paclitaxel is a well-recognized palliative treatment for high grade serous ovarian cancer. The response rates to weekly paclitaxel in a platinum-resistant setting are approximately 25–40%, with a progression-free survival of approximately 3–4 months [5, 14]. There have been multiple trials that have tried to combine novel anticancer drugs with weekly paclitaxel and these have led to licensing of the anti-VEGF antibody bevacizumab for the treatment of platinum-resistant ovarian cancer [5].

mTORC1 inhibitors have been licensed for use in breast cancer in combination with aromatase inhibitors [15] and as a single agent in renal cancer [16]. The first generation of mTOR inhibitors was developed as allosteric inhibitors of mTOR and do not target the mTORC2 component of the mTOR complex. In some tumor types, this leads to feedback re-activation of AKT observed as an increase in AKT phosphorylation [17]. Vistusertib is a dual mTORC1/2 inhibitor [12] that has completed phase I evaluation and is being tested in multiple cancer types [11]. The mechanism of action of mTOR inhibitors can be multifactorial: inhibiting growth, angiogenesis, increasing apoptosis and altering metabolism within cancer cells and we aimed to study these features [18].

We were able to inhibit signaling within the PI3K/mTOR pathway; in particular, mTORC1 (S6) and mTORC2 (AKT) by vistusertib alone and in combination with paclitaxel in ovarian carcinoma cell lines. Inhibition of the PI3K/mTOR pathway in combination with paclitaxel has been described previously [19–21].

Our results suggest that there is additive growth inhibition caused by the combination of paclitaxel and vistusertib in a panel of ovarian cancer cell lines. This is consistent with previous reports evaluating the combination of paclitaxel and mTOR inhibitors, which have shown additive/synergistic activity of the combination, depending on the methods used to evaluate growth inhibition [22, 23]. Our *in-vitro* studies used a panel of established ovarian cancer cell lines. It is possible organoid models using freshly isolated cells from patients will better reflect chemoresistance in patients.

*In vivo*, the molecular targets of mTORC1 and mTORC2 were found to be inhibited in tumors treated with vistusertib alone or in combination with paclitaxel, as shown by the reduced expression of pS6 and p-AKT levels, respectively. It was possible to show significant growth inhibition in the combination arm, when compared to vehicle control and no significant change in tumor growth was found in the vistusertib or paclitaxel treatment alone when compared with vehicle control, indicating that the combination treatment had significant anti-tumor effects in A2780CisR xenografts while the single agents did not. We used the A2780Cis xenograft model as we thought it reflected a platinum resistant state of patients with advanced ovarian cancer. There is growing evidence that patient derived xenografts can be used to test drug efficacy/resistance and could be used as alternative models. In the xenograft model studied there was increased apoptosis (cleaved caspase 3 positive cells) and necrosis (by hematoxylin & eosin staining) in tumors treated with a combination of vistusertib and paclitaxel when compared to vehicle control, indicating that the combination therapy induced tumor cell death. We have studied cleaved caspase as a marker of reflective of apoptosis. Other markers such as cleaved PARP or cleaved cytokeratin 18 could be used to further characterize this. Increases in apoptosis by the combination of paclitaxel and PI3K/mTOR inhibition in ovarian cancer models has been shown before [20]. At the time-point studied in this model, there was no significant decrease in angiogenesis (CD34+ stained vessels) or proliferation (Ki67 positive cells). Anti-angiogenic agents such as bevacizumab are licensed for use in ovarian cancer [5] and mTORC1 inhibitors are known to have anti-angiogenic effects.

It was possible to further show that reduced phospholipid metabolism and compromized bioenergetics occurred in tumors treated with the combination of vistusertib and paclitaxel and not in tumors treated with either of the single agents alone. Phospholipid compounds are major components of cell membranes and are also involved in the regulation of cell functions. Elevated levels of PC and choline kinase (a cytosolic enzyme that catalyzes the phosphorylation of choline to form PC) are found in cancer cells and tumors and are associated with cell proliferation and malignant transformation in addition to being regulated by several major signaling pathways such as the PI3K-AKT-mTOR pathways [24] . Inhibition of PI3K, AKT or mTORC1 resulted in reduced PC and choline kinase expression and activity in cancer cells [25–27]. These literature reports are consistent with our findings of decreased PC and choline kinase expression in ovarian carcinoma tumors in the combination arm but not in the vistusertib alone group at this time-point. Further studies will be required to examine whether these parameters have changed in the vistusertib group alone at a different time-point. Furthermore, increases in PC and PE are associated with rapid tissue growth or membrane synthesis [28], whereas GPC and glycerophosphoethanolamine are produced during membrane breakdown [29]. Our observation of decreased PC, PE and GPC levels in tumors following the combination treatment indicates reduced membrane turnover and is consistent with its delay in tumor growth when compared with vehicle controls. This study also demonstrates that the combination treatment leads to a decreased ATP pool, which is not observed in either of the single agents. These compromized bioenergetics could ultimately lead to cell death and tumor growth inhibition.

Though not studied in tumor tissue in patients, it was interesting to demonstrate, for the first time, an induction of p-AKT levels in PRP caused by paclitaxel and abrogation of this following the addition of vistusertib in ovarian cancer patients receiving paclitaxel and vistusertib. This provided proof-of-concept that it was possible to achieve drug concentrations in patients which do recapitulate signaling changes such as down-regulation on AKT signaling by vistusertib observed in preclinical models.

To conclude, it was possible to demonstrate additive growth inhibition across a panel of ovarian cancer cell lines. Both *in vitro* and *in vivo* studies suggest additive growth inhibition and this is being tested in clinical trials. The changes in signaling have been further validated in tumor tissue. The *in vivo* model studied showed significant increases in apoptosis and necrosis, compromized tumor bioenergetics and reduced membrane turnover, which is consistent with increased growth delay seen in the combination group. This suggests these metabolic and anti-tumor effects were caused by the combination over either single agents alone and this gives a mechanistic insight into the combination therapy. Part of this data was used as the basis for the ovarian cancer expansion of the TAX-TORC study (NCT02193633). Data generated from this study has led to the evaluation of the combination in a randomized phase II study in platinum- resistant ovarian cancer (OCTOPUS study).

## MATERIALS AND METHODS

### Cell lines and chemicals

All cell lines were obtained from ATCC, Teddington, UK or were available in labs within The Institute of Cancer Research, London, UK and short tandem repeat typed. Paclitaxel was purchased from Sigma and vistusertib was provided by AstraZeneca.

### Cell proliferation

The cell proliferation assay was determined by measuring the number of Hoechst-stained nuclei. Briefly, cells were seeded in 96-well plates (3000–5000 cells per well) and exposed to increasing concentrations of vistusertib and paclitaxel for 72 hours. Cells were fixed in 3.7% formaldehyde, stained with Hoechst 33258 (Invitrogen), and counted on CellInsight (Thermo Scientific). Cell numbers were normalized to day 0 and GI50 for each cell line was determined using Genedata Screener 12 software.

### Calculation of synergy score

A panel of 12 ovarian cancer cell lines, representing different subtypes of ovarian cancer, was screened to identify cell lines in which vistusertib synergizes with paclitaxel to inhibit proliferation. Cells were treated with increasing concentrations of vistusertib and paclitaxel or the combination in a 6 × 6 dosing matrix and cell number was measured after 5 days of treatment using SYTOX Green endpoint. Two-dimensional dose response matrix and curve fitting were processed in the combination extension of Genedata Screener 12™.

The combination was evaluated in a 6 × 6 dose matrix format, which allows the drug combination activity to be analysed over a wide concentration range. Using a quantitative synergy score based on the Loewe model of additivity [30], relative synergy scores were calculated.

### A2780CisR xenograft model

NCr female nude mice were injected subcutaneously in the flank with 5 × 10^6^ A2780CisR human cisplatin-resistant ovarian carcinoma cells. Tumor volume was calculated by measuring the length, width, and depth using callipers and the formula L × W × D × (π/6). Once an appropriate tumor volume (∼80 mm^3^) was established, mice were randomly divided into four groups (*n* = 9 in each group), they were treated for two weeks with: (i) paclitaxel alone at 20 mg/kg/1 day/week iv (P); (ii) vistusertib alone at 50 mg/kg/3 days/week po (A); (iii) a combination of paclitaxel at 20 mg/kg/1 day/week iv and vistusertib at 50 mg/kg/3 days/week po (A+P); (iv) vehicle (10% of DMSO) for 20 mg/kg/1 day/week iv and 50 mg/kg/3 days/week po (V). Three hours after the last dose at week 2 (day 9), tumors were excised for ^31^P-magnetic resonance spectroscopy (MRS) and western blotting analysis. Animals were treated in accordance with local and national research ethics committee requirements and in accordance with the United Kingdom Coordinating Committee on Cancer Research Guidelines for the Welfare of Animals in Experimental Neoplasia [31].

### *In vitro* ^31^P-MRS of A2780CisR tumor extracts

Freeze-clamped A2780CisR tumors were extracted using a dual phase method [32]. Neutralized extracts were freeze-dried and reconstituted in 700 µl D2O and 600 µl of this solution was then analyzed. Ethylenediamine tetra-acetic acid (EDTA; 50 µl, 60 mM) was added to chelate metals ions, and methylenediphosphonic acid (MDPA; 50 µl, 5 mM) added as a chemical shift and quantification reference. ^31^P-MRS spectra were acquired on a Bruker 500 MHz MR system (Bruker Biospin, Coventry, UK), using a power-gated composite pulse ^1^H decoupling sequence, 30°C flip angle, 5 s repetition delay, spectral width of 50 ppm and 32 K data points. Spectral processing and metabolite quantitation were performed as previously described [33].

### Immunohistochemistry

Expression of caspase-3 (apoptotic marker), CD34 (micro-vessel density) and ki67 (proliferation marker) were determined by immunohistochemistry, using the streptavidin-biotin peroxidase technique. Briefly, sections of 5 µm were de-paraffinized in xylene and rehydrated in different percentages of ethanol up to distilled water for 30 min. Antigen retrieval was performed by microwaving the sections in 10 mM sodium citrate buffer pH6 at 10 min intervals for a total of 20 min and cooling for 1h at room temperature (RT). Endogenous peroxidase activity was blocked by incubating the sections in a solution of 3% hydrogen peroxide for 20 min at RT. After washing in PBS (phosphate buffer saline), sections were incubated with the primary polyclonal rabbit anti-human caspase-3 (1:50, ABCAM ab2302), monoclonal rabbit anti-human CD34 (1:200, ABCAM ab81289), mouse monoclonal anti-human Ki67 (1:75 DAKO M7240), overnight at 4°C. The sections were washed with PBS and incubated with a biotinylated secondary antibody for 45 min, followed by an incubation with streptavidin-biotin horseradish peroxidase complex (DAKO) for another 45 min, at RT. Staining was carried out using a solution 3,3’-diaminobenzidine (DAB-Sigma), and lightly counter-stained with Harris’s hematoxylin.

Sections known to express high levels of caspase-3 (pancreas), CD34 (liver) and Ki67 (tonsil) were included as positive controls, while negative control slides were incubated with PBS. Immuno-stained slides were assessed by light microscopy and scored semi-quantitatively using Image J (1.50i). Cells positive for caspase-3 expression showed strong nuclear staining, were scored and averaged. Vascular structures with lumen were semi-quantified and were positive for CD34 as a brown staining with cytoplasmic distribution in endothelial cells. Ki67 staining index for each section was calculated as the percentage of positively stained tumor nuclei and the caspase-3 apoptotic index as a mean value of apoptotic cells and bodies in 3 randomly selected field of views.

### Western blotting

As described previously [34], protein expressions on tumor lysates were analysed by western blotting. 15ug tumor protein lysate was transferred onto Immobilon-P membranes (Millipore: Bedford, MA, USA) and the blots incubated with pS6 ribosomal protein (Cell Signalling 9205), p-AKT (Cell Signalling 9271) and Cho-K (Sigma HPA024153). b-actin (Cell Signalling 4967) was used as loading control. The membranes were then incubated with anti-rabbit secondary antibody (GE Healthcare, UK). Specific-binding antibody-target protein interactions were detected using enhanced chemiluminescence (Amersham Biosciences, UK) and exposure to X-OMAT Kodak (Kodak, USA) autoradiography film. For *in vitro* analysis cells were lysed in Pierce RIPA buffer (Thermo Scientific), supplemented with protease inhibitor cocktail (Roche), and PhosSTOP phosphatase inhibitor (Roche). Antibodies were diluted in 5% milk-PBS-Tween and signal detected using SuperSignal West Dura HRP substrate followed by visualization on a Syngene ChemiGenius Imager.

Antibodies used for p70S6K (CST 9202, 1:1000); p70S6K pT389 (CST 9205, 1:500); AKT (CST 9272, 1:1000), pAKT S473 (CST 9271, 1:500), S6 (CST 2317, 1:1000), pS6 S235/236 (CST 4858, 1:000), PDCD4 (CST 9535, 1:1000), vinculin (Sigma V9131, 1:5000).

### Human pharmacodynamic monitoring

Blood samples were collected after obtaining informed consent (The Institute of Cancer Research and The Royal Marsden Committee for Clinical Research: CCR no. 3667). Blood samples were obtained from patients with ovarian cancer at scheduled time-points: Pre-dose on cycle 1, day 1 (C1D1; pre), 4 h post-paclitaxel dose on cycle 1, day 1 (C1D1; 4 h), 4 h post-vistusertib on cycle 1, day 3 or 4 (C1D3/4; 4 h) and 4 h post-combination dose of paclitaxel and vistusertib on cycle 1 day 8 (C1D8; 4 h). All patients received 80 mg dose of paclitaxel, with vistusertib doses ranging from 25 – 100 mg on two schedules of 3 days on, 4 days off or 2 days on, 5 days off. Pharmacodynamic (PD) biomarkers of AKT signaling including p-AKT (Ser473 residue) were measured in PRP using previously published Mesoscale Discovery *(MSD®)* multiplex electrochemiluminescent immunoassays validated for Good Clinical Practice applications by The Institute of Cancer Research [35].

### Statistics

The tumor volume, immunohistochemical and metabolite data were expressed. As median and interquartile ranges (25% and 75%). Non-parametric Kruskal-Wallis test followed by Dunn’s multiple comparison test were used to compare the control and the treatment groups (GraphPad Prism 6, GraphPad Software Incorporated, USA). *p* < 0.05 was considered significant.

## Abbreviations

ATP: adenosine triphosphate
C: cycle
D: day
GPC: glycerophosphocholine
H: hour
IV: intravenously
PC: phosphocholine
PDCD4: programed cell death protein 4
PE: phosphoethanolamine
PRP: platelet-rich plasma
SEM: standard error of the mean
